# Presentation and Outcomes of Localized Immunoglobulin Light Chain Amyloidosis: 14‐Year Experience of an Academic Center

**DOI:** 10.1002/hon.70082

**Published:** 2025-04-13

**Authors:** Danai Dima, Utkarsh Goel, Fauzia Ullah, Beth Faiman, Diana Basali, Sandra Mazzoni, Louis S. Williams, Christy Samaras, Jason Valent, Faiz Anwer, Jack Khouri, Shahzad Raza

**Affiliations:** ^1^ Cleveland Clinic Taussig Cancer Center Cleveland Ohio USA; ^2^ University of Washington Fred Hutchinson Cancer Center Seattle Washington USA

**Keywords:** AL amyloidosis, amyloidosis, localized AL amyloidosis, localized amyloidosis, plasma cell disorder

## Abstract

Localized light chain amyloidosis (loc‐AL) is a rare disorder characterized by localized deposition of misfolded AL fibrils. There are limited data on patterns of disease presentation and long‐term outcomes. In this study, we retrospectively reviewed 146 patients with loc‐AL at our institution between January 1, 2010, and March 1, 2024. We excluded patients with evidence of systemic AL amyloidosis. We calculated local (PFS_L_) and systemic (PFS_s_) progression free survival (PFS) and overall survival (OS) using the Kaplan‐Meier method. We found that loc‐AL most commonly involved the respiratory (26%), gastrointestinal (17%), head and neck (17%) and genitourinary (10%) systems. Overall, 51% patients were asymptomatic at presentation, and 16% had a co‐existent autoimmune disease. First line management included observation (52%), surgical resection (39%), chemotherapy (3%), and radiation (2%). Most patients (59%) had a response with first‐line therapy. The median PFS_L_ was ∼15 years (10‐year PFS_L_ 68%), and median OS was not reached (10‐year OS 83%). None of the patients had progression to systemic amyloidosis. Seventeen patients had local recurrence and required second line therapy. In conclusion, loc‐AL has an excellent prognosis and does not progress to systemic AL amyloidosis. Observation and/or surgical removal are usually adequate first‐line approaches; however, a small proportion of patients can relapse locally requiring repeated interventions for symptom control.

## 1 Introduction

Amyloidosis is a rare group of disorders characterized by the production of abnormal protein misfolded into amyloid fibrils which further deposit in various organs causing varying degrees of impairment. There are several types of amyloidoses, based on the kind of misfolded protein and source of production [1]. The disease manifestations of amyloidosis are wide, ranging from systemic multi‐organ involvement to localized disease. Systemic amyloid light chain (AL) amyloidosis is the most common type of amyloidosis, in which non‐functional immunoglobulins are secreted by a malignant clone of plasma cells in the bone marrow, fold into amyloid fibrils and then get deposited in major organs like heart, kidneys, and peripheral nervous system, leading to significant morbidity and mortality [2, 3]. However, in rare cases, localized deposition of AL amyloid fibrils has been also observed in the absence of systemic malignant disease, with no evidence of bone marrow involvement and negative biomarkers in serum and urine ‐ an entity recognized as localized AL amyloidosis (loc‐AL) [4].

Due to the rare nature of this disease, the pathophysiology of loc‐AL is not well characterized. However, it is believed that the amyloid deposits in this case are produced by a clone of localized plasma cells or B‐cells in the setting of local inflammation [5]. Deposition of loc‐AL can be seen almost anywhere in the body and clinical symptoms depend on the location and the size of lesions [6, 7, 8, 9, 10, 11, 12, 13]. They can range from asymptomatic disease to severe manifestations most commonly due to mass effect of the AL amyloid deposits.

To date, three groups have described their extensive clinical experience (> 100 patients) with loc‐AL [14, 15, 16], however, our knowledge still remains limited, and the disease is poorly understood. In these studies, the respiratory tract and the genitourinary system have been identified as the most common sites associated with loc‐AL, in addition to smaller case series [10, 17, 18, 19]. In addition, patients were frequently diagnosed with concomitant autoimmune disorders. Overall, most patients with loc‐AL do not seem to progress to systemic AL amyloidosis. Life expectancy appears to be comparable to the general population, however, recurrence after initial treatment may be common and sometimes hard to manage even with repeated interventions, which can adversely impact quality of life [15]. Often, diagnosis of Loc‐AL and appropriate work up can be a challenge in routine practice, given a lack of clinical consensus about the timing and duration of follow‐up. In this study, we aimed to describe our institution's experience of patients with loc‐AL, including disease presentation patterns, management, and subsequent local and systemic progression.

## 2 Methods

After approval from the Institutional Review Board, we retrospectively reviewed consecutive adult patients diagnosed with amyloidosis who were treated at our institution between January 1, 2010, and March 1, 2024. Baseline demographic characteristics, mode of presentation, organ involvement, and treatment related information were collected via manual review of the electronic medical records.

The diagnosis of loc‐AL was made with a positive Congo red or Thioflavin S stains of the affected tissue samples, and typing was done with either immunohistochemical staining or mass spectrometry [20, 21]. Patients with evidence of systemic AL amyloidosis involving major organs, such as heart, kidney, liver, and nervous system in addition to presence of circulating monoclonal immunoglobulin on initial assessment were excluded. Patients with bone marrow and/or fat pad biopsies positive for AL amyloid were excluded from the present study. Additionally, patients with other types of localized amyloidosis such as transthyretin amyloidosis (ATTR) were also excluded. In cases of loc‐AL with concomitant presence of a monoclonal protein in the serum, systemic AL amyloidosis was thoroughly ruled out.

Assessments for response to therapy and local progression or local recurrence were determined by follow‐up clinical examinations, imaging studies (including endoscopy) and/or repeated biopsies. Based on response to therapy, loc‐AL was classified into improved, stable, or worsened in response to therapy. Progression to systemic AL amyloidosis was defined by the new detection of AL amyloid deposits in vital organs, fat pad and/or bone marrow in addition to detection of paraproteinemia in serum and/or urine along with monoclonal plasma cells in the bone marrow. Some patients (mainly asymptomatic) did not have follow up imaging for response assessment, therefore these were coded as non‐evaluable for response.

Local progression free survival (PFS_L_), systemic progression free survival (PFS_S_), time to progression (TTP), and overall survival (OS) were calculated using the Kaplan‐Meier method and differences between groups assessed using the log‐rank test. PFS_L_ was calculated from diagnosis until local progression or recurrence or death. PFS_S_ was calculated from diagnosis of loc‐AL until first documented progression to systemic AL amyloidosis or death. TTP was calculated from diagnosis until local progression and deaths during this time frame were censored. OS was calculated from loc‐AL diagnosis to death due to any cause. Differences between groups were evaluated using chi‐squared or Fisher's exact tests for categorical variables, or Kruskal‐Wallis test for continuous variables. The median follow up was calculated using the reverse Kaplan‐Meier estimator method. All statistical tests were two sided and *p*‐values of less than 0.05 were considered statistically significant. All statistical analyses were performed using R version 4.3.1.

## 3 Results

### 3.1 Baseline Patient Characteristics and Presentation Patterns

Of the 3146 patients with amyloidosis treated at Cleveland Clinic, 146 (5%) had loc‐AL and were included in the study. Baseline characteristics at loc‐AL diagnosis are described in Table 1. The median age at diagnosis was 67 (range 18–92) years. The most common sites involved with loc‐AL were the respiratory system (*n* = 38, 26%), gastrointestinal (GI, *n* = 25, 17%), head and neck region (*n* = 25, 17%), and genitourinary (GU) system (*n* = 14, 10%). At presentation, 71 (49%) patients were symptomatic from loc‐AL. Clinical manifestations varied based on the type of tissue involved and anatomical location (Table S1). Systems most frequently presenting with symptoms were the upper respiratory tract (7/8 patients, 88%), head and neck (20/25 patients, 80%) and soft tissue (12/17 patients, 71%, Table 2). Sites where loc‐AL was most frequently asymptomatic included the breast (2/10 patients, 20%), the lower respiratory tract (7/30 patients, 23%) and the GI system (8/25 patients, 32%). At these locations, most loc‐AL lesions were found incidentally on imaging for an unrelated condition, or routine screening mammograms.

**TABLE 1:** Baseline patient characteristics at loc‐AL diagnosis.

Characteristics	*N* = 146 (100%)
Age, median (range)	67 (18–92) years
Sex	66 (45%)
Race	
White	122 (84%)
Black	21 (14%)
Hispanic	3 (2%)
Organ/System involvement	
Upper & lower respiratory	38 (26%)
Gastrointestinal	25 (17%)
Head & neck	25 (17%)
Soft tissue	17 (12%)
Genitourinary	14 (10%)
Skin	12 (8%)
Breast	10 (7%)
Central nervous system	3 (2%)
Cardiac	2 (1%)
Light chain type	
Kappa	68 (47%)
Lambda	56 (38%)
Polyclonal	22 (15%)
Serum IFE	
Negative	116 (79%)
Positive	30 (21%)
Heavy chain	
IgG/IgA/IgM/None	21 (14%)/2 (2%)/6 (4%)/1 (1%)
Light chain	
Kappa/Lambda	16 (11%)/14 (10%)
LC same as loc‐AL	18 (12%)
Abnormal FLC ratio	18 (12%)
Bone marrow biopsy	
Negative	71 (49%)
Not done	75 (51%)
Fat pad biopsy	
Negative	19 (13%)
Not done	127 (87%)
Cardiac imaging (echo and/or MRI)	
Negative	107 (73%)
Not done	39 (27%)
Cardiac biomarkers	
Negative	90 (62%)
Positive	39 (27%)
Not done	17 (12%)
Concomitant autoimmune disease	23 (16%)
Rheumatoid arthritis	7 (5%)
Sjogren's syndrome	6 (4%)
Psoriasis	3 (3%)
Scleroderma	3 (3%)
Sarcoidosis	2 (2%)
Lupus	2 (2%)
Mixed connective tissue disease	1 (1%)
Lichen planus	1 (1%)
Celiacs	1 (1%)
Clinical presentation	
Symptomatic	71 (49%)
Asymptomatic	75 (51%)

Abbreviations: FLC, free light chains; IFE, immunofixation; Ig, immunoglobulin; LC, light chain; loc‐AL, localized AL amyloidosis; MALT, mucosa associated lymphoid tissue lymphoma; MRI, magnetic resonance imaging.

**TABLE 2:** Detailed organ/system involvement and clinical presentation (*N* = 146).

Organ involvement	Age (median, range)	Sex Male (*N* ‐ %)	Main symptoms	Asymptomatic *(N* ‐ %)	Positive serum IFE (*N* ‐ %)	Matched sFLC & loc‐AL (*N* ‐ %)	Abnormal FLC ratio (*N* ‐ %)	Autoimmune disease (*N* ‐ %)
Gastrointestinal (*n* = 25)								
Esophagus, stomach, *n* = 6	69 (46–76)	3 (50)	Dysphagia, pain, GI bleed	2 (33)	0 (0)	0 (0)	3 (50)	0 (0)
Small bowel, colon, *n* = 18	67 (44–83)	10 (56)	GI bleed, early satiety, pain	14 (78)	6 (33)	3 (17)	0 (0)	3 (17) RA, lupus, Psoriasis
Gallbladder, *n* = 1	81	0 (0)	N/A	1 (100)	0 (0)	0 (0)	0 (0)	0 (0)
Respiratory (*n* = 38)								
Upper (subglottic area, larynx, vocal cords), *n* = 8	58 (35–71)	3 (38)	Hoarseness, dyspnea	1 (13)	3 (38)	2 (25)	0 (0)	1 (13) Sjogren's
Lower (trachea, lungs), *n* = 30	69 (28–86)	11 (37)	Dyspnea	23 (77)	8 (27)	5 (17)	4 (13)	7 (23)
Soft tissues, breast, skin (*n* = 39)								
Soft tissues, *n* = 17	69 (54–92)	9 (53)	Carpal tunnel, Trigger finger, Pain	4 (24)	2 (12)	2 (12)	2 (12)	4 (24) Sjögren's, RA, scleroderma, psoriasis
Breast, *n* = 10	66 (44–73)	0 (0)	Discomfort	8 (80)	2 (20)	1 (10)	2 (20%)	3 (30) Sjögren's, RA
Skin, *n* = 12	68 (27–80)	5 (42)	Rash, pruritus	7 (58)	3 (25)	2 (17)	2 (17)	1 (8) RA
Head & neck (*n* = 25)								
Eye (conjunctiva, orbit, eyelid), *n* = 16	68 (18–85)	8 (50)	Pain/discomfort Blepharoptosis Irritation, edema	1 (6)	1 (6)	1 (6)	1 (6)	2 (13) Lupus, sarcoidosis
Oropharyngeal, *n* = 8	55 (35–69)	4 (50)	Pain, epistaxis	4 (50)	1 (13)	1 (13)	0 (0)	0 (0)
Ear (auditory canal), *n* = 1	80	1 (100)	N/A	1 (100)	0 (0)	0 (0)	0 (0)	1 (100), RA
Genitourinary (*n* = 14)								
Bladder, urethra, Ureter, *n* = 11	60 (22–85)	8 (73)	Hematuria Difficulty urinating	2 (18)	2 (18)	0 (0)	1 (9)	1 (9) Psoriasis
Prostate, *n* = 3	73 (72–75)	3 (100)	N/A	3 (100)	1 (33)	1 (33)	1 (33)	0 (0)
Other (*n* = 5)								
Cardiac (MV, atrial appendage), *n* = 2	66 (64–68)	0 (0)	N/A	2 (100)	1 (50)	0 (0)	1 (50)	0 (0)
CNS, *n* = 3	62 (40–74)	1 (33)	Spastic Ity	2 (67)	0 (0%)	0 (0)	0 (0%)	0 (0)

Abbreviations: FLC, free light chains; GI, gastrointestinal; MALT, mucosa‐associated lymphoid tissue lymphoma mitral valve; N/A, not applicable; RA, rheumatoid arthritis; SCC, squamous cell carcinoma; sFLC, indicates serum free light chains.

Bone marrow biopsy, fat pad biopsy and cardiac imaging (echocardiogram and/or cardiac MRI) were obtained in 71 (49%), 19 (13%) and 107 (73%) patients, respectively. All bone marrow and pad biopsies were negative for AL amyloid; in addition, none of the bone marrow biopsies revealed monoclonal plasma cells. A serum monoclonal protein (sMP) was obtained in all patients and was detected positive in only 30 (21%) patients, 12 of whom had serum light chain restriction different from the loc‐AL light chain type. Of the 30 patients with positive sMP, 21 had IgG isotype, 6 had IgM, 2 had IgA and 1 had light chains only. Most patients with a positive sMP had respiratory involvement of loc‐AL (11/30 patients, 37%) followed by GI involvement (6/30 patients, 20%). An abnormal serum free light chain ratio was reported in 18 (12%) patients, and six of these patients also had a positive sMP. Among all patients at diagnosis, 39 (27%) had at least one abnormal cardiac biomarker (NT‐proBNP or high sensitivity troponin T). Of these 39 patients, 33 (85%) had no evidence of cardiac amyloid involvement on cardiac imaging (cardiac imaging not done at diagnosis for the remaining 6 patients).

Twenty‐three (16%) patients had coexistent autoimmune disease, with three of them having more than one type of autoimmune disease. Concurrent autoimmune disease was most common in patients with breast loc‐AL involvement (3/10 patients, 30%), lower respiratory tract involvement (8/30 patients, 27%) and soft tissue involvement (4/17 patients, 23%). Most common types of autoimmune diseases were Sjögren syndrome in seven patients and rheumatoid arthritis in six cases. Sixteen patients (11%) had a co‐existent malignancy that was not plasma cell‐related (Table 1).

### 3.2 First‐Line Management Approaches

First line management approaches included: observation with or without supportive care (76 patients, 52%), surgical resection (57 patients, 39%), radiation therapy (3 patients, 2%), systemic chemotherapy (5 patients, 3%), systemic steroids (5 patients, 3%), topical steroids (4 patients, 3%) and botulinum toxin injection (1 patient, 1%). Overall, 86 (59%) patients improved, 48 (33%) remained stable, nine (6%) were non‐evaluable, and three (2%) deteriorated with first line therapy. Among symptomatic patients only, 56 (79%) improved, 13 (18%) remained stable, and two (3%) deteriorated. Details of first line therapies per organ system and responses are described in Table 3. Of the five patients who received systemic chemotherapy, 4 received plasma cell‐directed therapy and maintained stable disease based on follow‐up imaging. The fifth patient received rituximab for loc‐AL associated with mucosa‐associated lymphoid tissue (MALT) with worsening of loc‐AL despite therapy.

**TABLE 3:** First‐line therapeutic strategies and outcomes per organ system involved (*N* = 146).

Organ	Observation (*N* ‐ %)	Surgical removal (*N* ‐ %)	XRT (*N* ‐ %)	Systemic Steroids (*N* ‐ %)	Systemic chemo (*N* ‐ %)	Topical steroids (*N* ‐ %)	Improved/Stable/NE (*N* ‐ %)	Local progression (*N* ‐ %)	Received further RX (*N* ‐ %)
Gastrointestinal (*n* = 25)									
Esophagus, stomach, *n* = 6	5 (83)	1 (17)	—	—	—	—	2 (33)/3 (50)/1 (17)	—	—
Bowel, gallbladder *n* = 19	16 (84)	2 (11)	—	—	1 (5)	—	7 (37)/5 (26)/7 (37)	1 (5)	1 (5)
Respiratory (*n* = 38)									
Upper respiratory, *n* = 8	2 (25)	4 (50)	1 (12.5)	1 (12.5)	—	—	6 (75)/2 (25)/‐	3 (37.5)	3 (37.5)
Lower respiratory, *n* = 30	22 (73)	4 (13)	1 (3)	2 (6)	3 (10)	—	9 (30)/21 (70)/‐	3 (10)	3 (10)
Soft tissue, skin (*n* = 39)									
Soft tissues, *n* = 17	6 (35)	11 (65)	—	—	—	—	13 (76)/3 (18)/‐	1 (6)	1 (6)
Breast, *n* = 10	7 (70)	3 (30)	—	—	—	—	4 (40)/6 (60)/‐	2 (20)	2 (20)
Skin, *n* = 12	7 (58)	2 (17)	—	2 (17)	—	2 (17)	(75)/3 (25)/‐	1 (8)	1 (8)
Head & neck (*n* = 25)									
Ear/Eye, *n* = 17	2 (12)	14 (82)	—	—	—	1 (6)	15 (88)/1 (6)/‐	1 (6)	1 (6)
Oropharyngeal, *n* = 8	4 (50)	4 (50)	—	—	—	—	6 (75)/1 (12.5)/‐	1 (12.5)	1 (12.5)
Genitourinary (*n* = 14)									
Bladder, urethra, ureter, *n* = 11	5 (45)	6 (55)	—	—	—	—	9 (82)/2 (18)/‐	4 (36)	4 (36)
Prostate, *n* = 3	—	3 (100)	—	—	—	—	3 (100)/‐/‐	—	—
Other (*n* = 5)									
Cardiac, *n* = 2	—	2 (100)	—	—	—	—	2 (100)/‐/‐	—	—
CNS, *n* = 3	—	1 (33)	1 (33)	—	1 (33)	—	1 (33)/2 (67)/‐	—	—

Abbreviations: CNS, central nervous system; NE, non‐evaluable; RX, treatment; XRT, indicates radiation therapy.

### 3.3 Progression‐Free and Overall Survival After First Line Therapy

**FIGURE 1:**
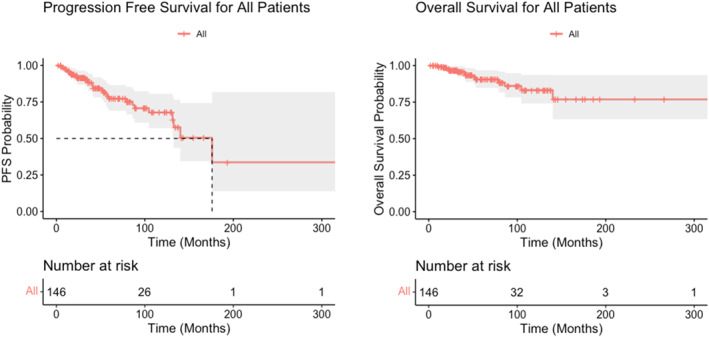
Progression free survival (PFS_L_) and overall survival (OS) for the entire cohort.

The median follow‐up for the entire cohort was 49 months (95% CI: 40–61 months). By the end of follow‐up, 17 patients had local progression or recurrence, and 13 patients had died. The median PFS_L_ was 176 months (∼15 years) (95% CI: 133‐ not reached, NR). The 1‐year, 2‐year, 5‐year and 10‐year PFS_L_ rates (95% CI) were 96% (93%–100%), 92% (87%–96%), 77% (69%–87%), and 68% (57%–81%) respectively. The 1‐year, 2‐year, 5‐year, and 10‐year progression free (TTP) rates (95% CI) were 97% (94%–100%), 95% (91%–99%), 86% (79%–94%), and 83% (75%–93%) respectively. The median OS was NR, and 1‐year, 2‐year, 5‐year, and 10‐year OS rates (95% CI) were 99% (98%–100%), 97% (94%–100%), 91% (85%–97%), and 83% (74%–94%) respectively (Figure 1). No PFS_L_ differences were found in the overall cohort when stratified by site of loc‐AL involvement. However, patients with respiratory loc‐AL involvement had an inferior PFS_L_ as compared to patients with non‐respiratory sites of involvement (median PFS_L_ 131 vs. 176 months, *p* = 0.049). Similarly, patients with GU loc‐AL involvement had an inferior PFS_L_ as compared to patients with non‐GU sites of involvement (median PFS_L_ 56 vs. 176 months, *p* = 0.014). PFS_L_ curves by site of involvement are described in Figure S1. None of the patients in our cohort had progression to systemic amyloidosis.

### 3.4 Outcomes of Second Line Therapy

Of the 17 patients who received second line therapy for loc‐AL, 11 patients had an improvement with therapy while the remaining six had stable disease. Six patients had further recurrence or progression and received third line therapy for loc‐AL. The most common sites requiring repeated interventions were respiratory (*n* = 6), followed by GU (*n* = 4) (Table S2).

## 4 Discussion

In this study, we described the sites of involvement, presentation, patterns of care, and long‐term outcomes of 146 patients with loc‐AL treated at our institution. Loc‐AL was most seen in respiratory, GI, head & neck, and GU systems in our cohort. Around half of all patients were asymptomatic, and 16% had a co‐existing autoimmune disease. The choice of first line therapy varied based on the organ system and extent of loc‐AL involvement, and most patients had improvement in disease with first line therapy. A small fraction (12%) required second line therapy, with most patients responding to second line therapy. Loc‐AL generally had a favorable prognosis, with > 90% patients alive and local progression‐free at 5 years. Importantly, none of the patients had progression to systemic AL amyloidosis.

Our findings add to the limited literature on the presentation and outcomes of patients with loc‐AL. Three previous groups have found loc‐AL to be present most commonly in the respiratory, skin/soft tissue, and GU systems, similar to the findings of our study [14, 15, 16]. The most common first line approaches were observation and excision, again consistent with our findings. For patients who required intervention (excision, radiation, etc.), the vast majority demonstrated a response. Prior case series have also reported favorable outcomes with localized interventions [22, 23, 24, 25, 26]. Given the rarity of loc‐AL, significant variability in the symptoms and sites of presentation, and variability in patterns of recurrence, it is challenging to reliably compare the effectiveness of the different types of interventions (excision, radiation therapy, or systemic therapy). As a general principle, excision, when feasible and relatively low risk (based on location of tissue), might be considered first, especially when loc‐AL is in proximity to a radiosensitive healthy area that is susceptible to damage from radiation; therefore, there is a high risk of significant radiation‐related adverse effects. The decision about the choice of first line and subsequent interventions should needs to be individualized according to the site of presentation, severity of symptoms, and patient characteristics. In this context, a multidisciplinary discussion might be valuable in discussing the risks and effectiveness of each modality with the patient.

All patients (100%) in our study had loc‐AL diagnosis confirmed with amyloid typing with either immunohistochemical staining, or mass spectrometry, like the study by Basset et al. In contrast, Kourelis et al. and Mahmood et al. had reliable amyloid typing available for 15% and 41% of all loc‐AL cases, respectively.

A significant proportion of our patients had coexistent autoimmune disorders primarily Sjögren syndrome and rheumatoid arthritis, both of which have been previously described in patients with loc‐AL as well as patients with lymphoproliferative disorders such as mucosa‐associated lymphoid tissue lymphoma [MALT] or marginal zone lymphoma. Notably, some of the patients in our cohort had more than one autoimmune disorder and two patients had MALT lymphoma associated with loc‐AL production [27, 28, 29, 30, 31, 32, 33]. Chronic antigenic exposure leading to autoimmune triggering has been hypothesized as a possible underlying mechanism. Of note, systemic therapy was not frequently utilized in our patient cohort; however, it is reasonable to consider it in the setting of a lymphoproliferative disorder or generalized autoimmune disease associated with loc‐AL production, to primarily treat these underlying processes as indicated by specific disease guidelines in an effort to also halt the loc‐AL production.

Previous studies have reported 17%–44% of all patients with loc‐AL that had a local recurrence. These rates are higher than the 12% of patients in our cohort who had a local recurrence, in spite of similar follow‐up periods in our study compared to previous studies. However, sites of local recurrence were similar among all studies mainly including the gastrointestinal and respiratory tracts. None of the patients in our study had progression of loc‐AL to systemic AL amyloidosis, consistent with rare systemic progression events reported in previous studies (0%–1%). Kourelis et al. posited that since loc‐AL by definition, is AL amyloid production due to a localized plasma cell clone, systemic progression might be a misnomer, and patients with loc‐AL who later experienced progression to systemic AL amyloidosis might have been misdiagnosed systemic AL amyloidosis cases in the first place. Indeed, in our study, when the presence of systemic AL amyloidosis was extensively ruled out at diagnosis/exclusion, none of the patients had a “systemic progression” to systemic AL amyloidosis. Finally, we observed an excellent overall survival in our cohort, consistent with 5‐year OS rates of ≥ 90% described in previous loc‐AL cohorts.

Overall, based on our results and findings from prior studies it is evident that early recognition, and longitudinal follow‐up are important in patients with loc‐AL for early intervention in symptomatic cases and prompt diagnosis of recurrence, especially in patients with GU or respiratory involvement to avoid excess morbidity. Given the heterogeneity of organ site involvement, most cases are diagnosed by non‐hematology specialists. Consultation with a hematology physician for complete work up to rule out systemic disease is crucial. Serum and urine screening immunofixation, and free light chains should always be obtained. Based on the results, bone marrow biopsy and fat pad biopsies can be considered. The latter are particularly relevant in cases where a concurrent monoclonal paraprotein is also identified (especially if light chain restriction of loc‐AL is the same as the serum paraprotein). If all tests are negative, no further work up is mandated. In terms of surveillance, this can be determined by the primary hematologist based on the severity of symptoms and location of localized disease. Given prior reports of potentially lower overall survival of patients with lung loc‐AL, these patients can be more rigorously monitored even if asymptomatic.

Our study is prone to several limitations inherent to its retrospective nature, and data being from a single institution, which could bias our results in terms of patient population and treatment patterns. Follow up intervals, follow up imaging, and treatments offered were not standardized, limiting comparison of sites of loc‐AL involvement in terms of response to therapy. Further, statistical comparison of presentations and outcomes was limited by a relatively small sample size.

## 5 Conclusion

Our study describes the presentation and treatment patterns of patients with loc‐AL. Loc‐AL is a rare type of amyloidosis and could be detected incidentally as around half of all patients are asymptomatic at diagnosis. Observation and/or surgical removal are usually adequate initial approaches to manage loc‐AL, however, a small percentage of patients can relapse locally requiring repeated interventions for symptom control. Choice of treatment must be individualized based on the site of organ involvement, extent of loc‐AL, and symptom burden. Overall, loc‐AL rarely progresses to systemic AL amyloidosis, especially if systemic disease is ruled out thoroughly at the time of diagnosis. Referral to a hematologist is important to rule out systemic disease involvement.

## Author Contributions

Conception and design: D.D, S.R. Provision of study materials or patients: All authors. Collection and assembly of data: All authors. Data analysis and interpretation: D.D., U.G., S.R. Manuscript writing: All authors. Final approval of manuscript: All authors.

## Disclosure

Faiz Anwer: Research Funding and Consultancy for BMS, Janssen, Allogene Therapeutics. Shahzad Raza: Advisory board for Kite Pharma, Pfizer and Prothena biosciences. Jason Valent: Research Funding from Alexion, AstraZeneca Rare Disease. Louis Williams: Consulting for BMS, Janssen, Abbvie. Jack Khouri: Consultancy for Janssen.

## Ethics Statement

This study was IRB approved and was conducted under the Declaration of Helsinki.

## Consent

Patient consent was not required per retrospective nature and IRB approval.

## Conflicts of Interest

The authors declare no conflicts of interest.

### Permission to Reproduce Material From Other Sources

Materials are original and not reproduced from other sources.

### Peer Review

The peer review history for this article is available at https://www.webofscience.com/api/gateway/wos/peer-review/10.1002/hon.70082.

## Supporting information

Supporting Information S1

## Data Availability

Data are available upon reasonable request to the corresponding author.
