# Supplementary material for: Presentation and Outcomes of Localized Immunoglobulin Light Chain Amyloidosis: 14‐Year Experience of an Academic Center
Source: Hematol Oncol. 2025 Apr 13;43(3):e70082. doi: 10.1002/hon.70082 (PMC11993898; doi:10.1002/hon.70082)

**Table S1.** Baseline patient characteristics per anatomical structure involved (Total N = 146 patients).

|  | **Gastrointestinal**  **(n=25)** | **Upper & lower Respiratory**  **(n=38)** | **Soft Tissue, Skin, Breast**  **(n=39)** | **Head & Neck**  **(n=25)** | **Genitourinary**  **(n=14)** | **Other**  **(n=5)** |
| --- | --- | --- | --- | --- | --- | --- |
| **Age, median (range)** | 68 (44-83) | 66 (28-86) | 69 (27-92) | 62 (18-85) | 68.5 (22-85) | 64 (40-74) |
| **Gender, male** | 13 (52) | 14 (37) | 14 (36) | 13 (52) | 11 (79) | 1 (20) |
| **Light Chain Type** |  |  |  |  |  |  |
| Kappa | 9 (36) | 21 (55) | 22 (56) | 12 (48) | 3 (21) | 1 (20) |
| Lambda | 10 (40) | 15 (40) | 8 (21) | 9 (36) | 10 (71) | 4 (80) |
| Polyclonal | 6 (24) | 2 (5) | 9 (23) | 4 (16) | 1 (7) | 0 (0) |
| **Serum IFE** |  |  |  |  |  |  |
| Negative | 19 (76) | 27 (71) | 32 (82) | 23 (92) | 11 (79) | 1 (20) |
| Positive | 6 (24) | 11 (29) | 7 (18) | 2 (8) | 3 (21) | 1 (20) |
| LC same as loc-AL | 3 (12) | 7 (18) | 5 (13) | 2 (8) | 1 (7) | 0 (0) |
| **Abnormal FLC ratio** | 4 (16) | 4 (11) | 5 (13) | 1 (4) | 2 (14) | 1 (20) |
| **Bone Marrow biopsy** |  |  |  |  |  |  |
| Negative | 11 (44) | 20 (53) | 21 (54) | 11 (44) | 6 (43) | 2 (40) |
| Not done | 14 (56) | 18 (47) | 18 (46) | 14 (56) | 9 (64) | 3 (60) |
| **Fat pad biopsy** |  |  |  |  |  |  |
| Negative | 3 (12) | 7 (18) | 5 (13) | 3 (12) | 1 (7) | 0 (0) |
| Not done | 22 (88) | 31 (82) | 34 (87) | 22 (88) | 0 (0) | 5 (100) |
| **Concomitant AD** | 3 (12) | 8 (21) | 8 (21) | 3 (12) | 1 (7) | 0 (0) |
| **Concomitant malignancy** | 1 (4) | 6 (16) | 4 (10) | 1 (4) | 2 (14) | 1 (20) |
| **Clinical Presentation** |  |  |  |  |  |  |
| Asymptomatic | 17 (68) | 24 (63) | 19 (49) | 6 (24) | 5 (36) | 4 (80) |
| Symptomatic | 8 (32) | 14 (37) | 20 (51) | 19 (76) | 9 (64) | 1 (20) |

Abbreviations: IFE indicates immunofixation; LC, light chain type; loc-AL, localized AL amyloidosis; AD, autoimmune disease.

| **Organ** | **Observation**  **(N - %)** | **Surgical Removal**  **(N - %)** | **XRT**  **(N -%)** | **Systemic Chemo**  **(N - %)** | **Topical Steroids**  **(N - %)** | **Other**  **(N - %)** | **Improved / Stable**  **/ NE (N - %)** | **Local Progression**  **(N - %)** | **Received Further RX**  **(N - %)** | **Type of RX** |
| --- | --- | --- | --- | --- | --- | --- | --- | --- | --- | --- |
| **Gastrointestinal (n=1)** |  |  |  |  |  |  |  |  |  |  |
| Bowel, n=1 | **-** | **-** | **-** | 1 (100), Dara |  | **-** | 1 (100) / **-** / **-** | **-** | **-** | **-** |
| **Respiratory (n=6)** |  |  |  |  |  |  |  |  |  |  |
| Upper Respiratory (Larynx, vocal cord, n=3 | **-** | 1 (33) | 1 (33) | **-** | 1 (33) | **-** | 2 (67) / 1 (33) / **-** | 1 (33) | 1 (33) | Steroid injections |
| Lower Respiratory (lung), n=3 | 2 (67) | **-** | 1 (33) | **-** | **-** | **-** | 1 (33) / 2 (67) / **-** | **-** | **-** | **-** |
| **Soft tissue, Skin (n=4)** |  |  |  |  |  |  |  |  |  |  |
| Soft tissues, n=1 | **-** | **-** | **-** | 1 (100),  Dara-VCd | **-** | **-** | 1 (100) / **-** / **-** | 1 (100) | 1 (100) | Dara, ASCT |
| Breast, n=2 | 1 (50) | 1 (50) | **-** | **-** | **-** | **-** | 1 (50) / 1 (50) / **-** | 1 (50) | 1 (50) | Excision |
| Skin, n=1 | **-** | 1 (100) | **-** | **-** | **-** | **-** | 1 (100) / **-** / **-** | 1 (100) | 1 (100) | Excision |
| **Head & Neck (n=2)** |  |  |  |  |  |  |  |  |  |  |
| Eye, n=1 | **-** | 1 (100) | **-** | **-** | **-** | **-** | 1 (100) / **-** / **-** | **-** | **-** | **-** |
| Oral cavity, n=1 | **-** | **-** | 1 (100) | **-** | **-** | **-** | 1 (100) / **-** / **-** | **-** | **-** | **-** |
| **Genitourinary (n=4)** |  |  |  |  |  |  |  |  |  |  |
| Bladder, Ureter, n=4 | **-** | 2 (50) | **-** | **-** | **-** | 3 (75%)  doxycycline,1 (25) stent | 1 (25) / 3 (75) / - | 2 (50) | 2 (50) | Excision, stents |

**Table S2.** Second and Third-Line Therapies and Outcomes of Patients who Progressed after First-Line Strategies (N=17).

Abbreviations: XRT indicates radiation therapy; NE, non-evaluable; RX, treatment; Dara-VCd, daratumumab, bortezomib, cyclophosphamide, and dexamethasone; Dara, daratumumab; ASCT, autologous stem cell transplantation.

**Figure S1**. Local progression free survival based on site of loc-AL involvement. Of note, none of the patients with cardiac loc-AL (n=2) had local progression or recurrence.


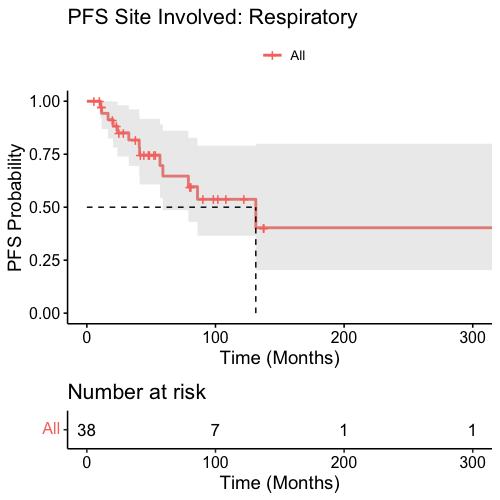

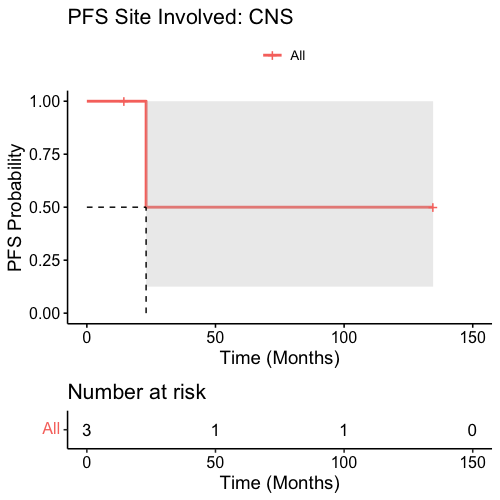

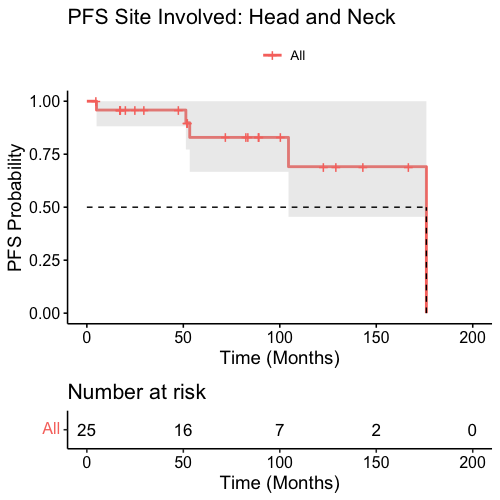


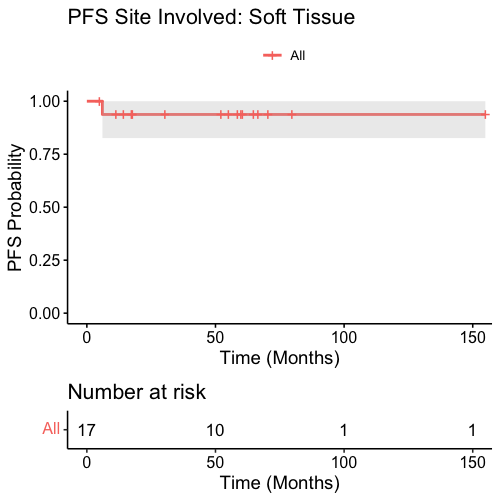

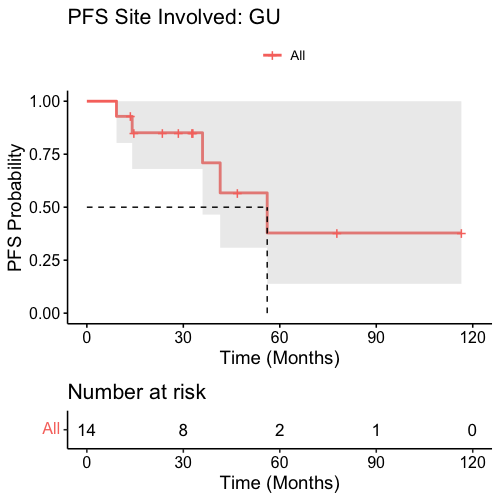

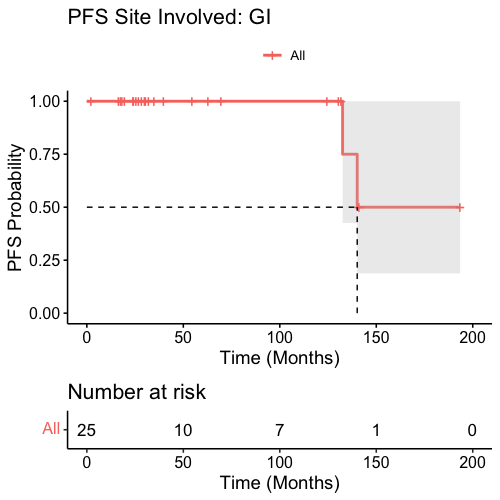


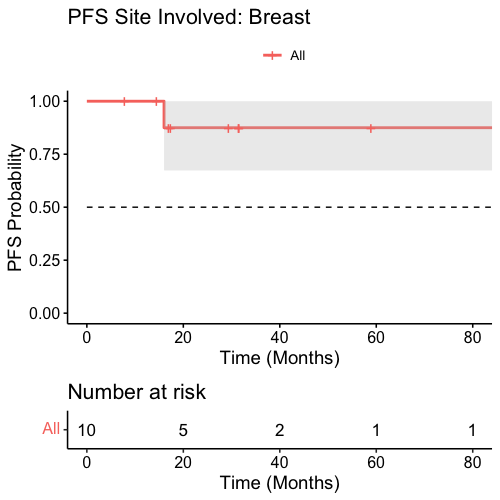

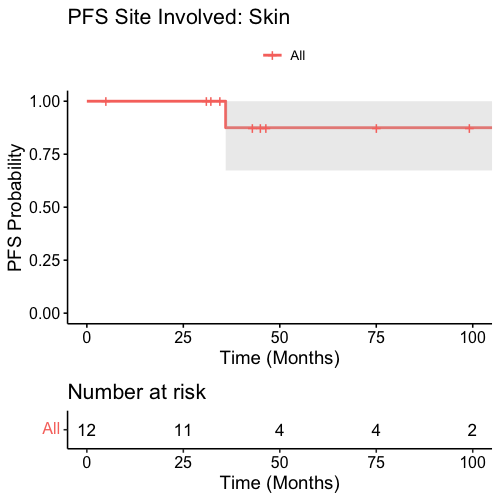

Supplement: Supplementary file 1 — Supporting Information S1 [file HON-43-e70082-s001.docx]
